# Supplementary figures and images for: The development of hepatic steatosis depends on the presence of liver-innervating parasympathetic cholinergic neurons in mice fed a high-fat diet
Source: PLoS Biol. 2024 Oct 22;22(10):e3002865. doi: 10.1371/journal.pbio.3002865 (PMC11530026; doi:10.1371/journal.pbio.3002865)

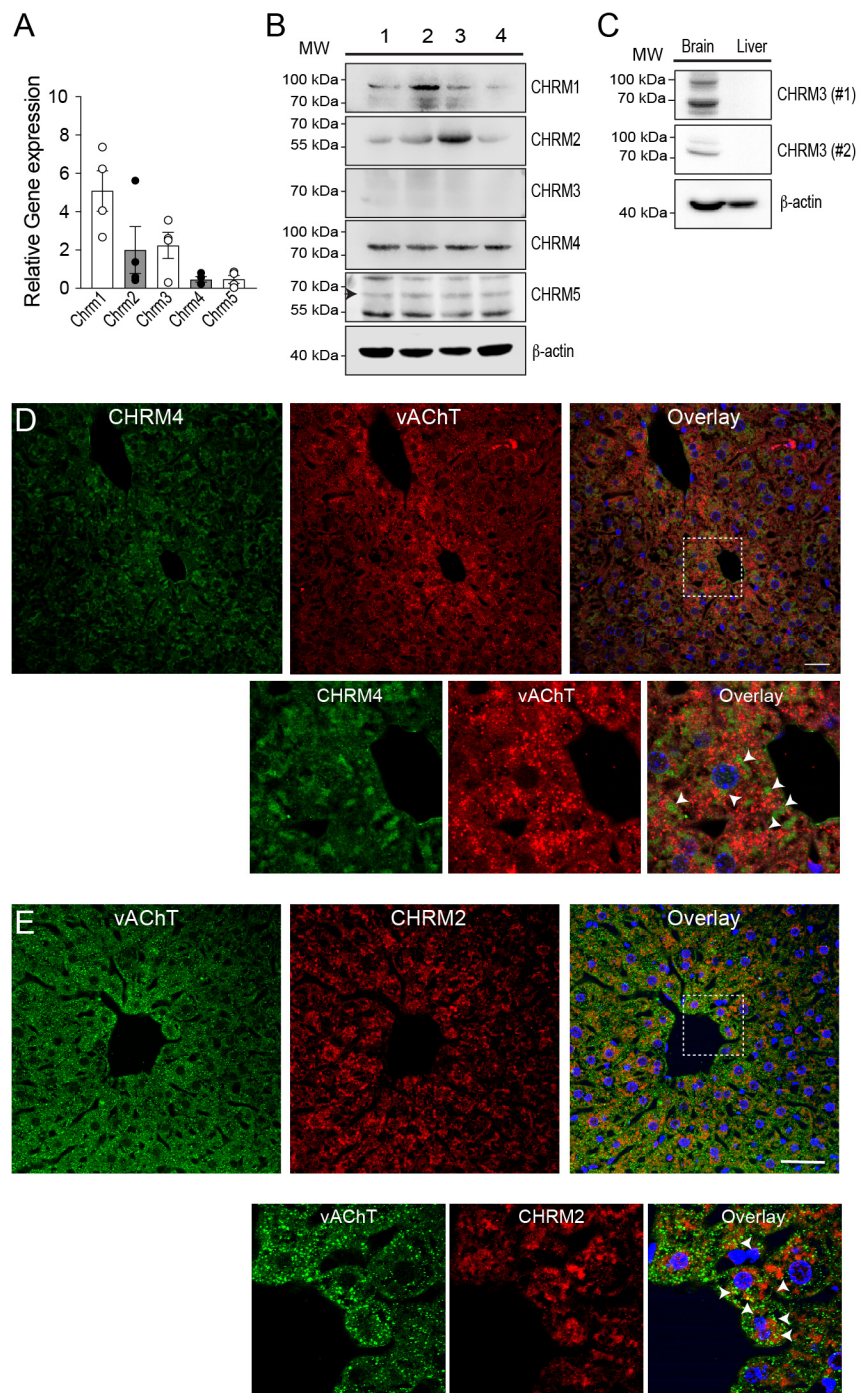

S1 Figure

Supplement: S1 Fig — (A) Graph showing mRNA expression of Chrm1, 2, 3, 4, and 5 in mouse liver samples. (B) Images of western blot analysis of liver homogenates showing the expression of CHRM1-5, except for CHRM3. (C) Western blot images showing the expression of CHRM3 in the brain, but not the liver samples. The antibodies used in this study were Alomone Labs (AMR 006) and Abcam (ab87199). (D) Images of confocal fluorescence microscopy showing the expression of CHRM4 and vAChT in the liver parenchyma (upper panel). Scale bar, 25 μm, bottom panel: Higher magnification view of the white dotted square. CHRM4s were found adjacent to vAChT-positive nerve terminals (white arrows). Blue: nucleus staining with DAPI. Scale bar, 10 μm. (E) Images of confocal fluorescence microscopy showing the expression of CHRM2 in hepatocytes. The vAChT-positive parasympathetic nerve terminals were adjacent to CHRM2. Scale bar, 50 μm, bottom panel: Higher magnification view of the white dotted square. Arrowheads represent examples of cholinergic synapses on hepatocytes. The data supporting the graphs shown in the figure (S1A Fig) are available in the S2 Data file. (PDF) [file pbio.3002865.s001.pdf]

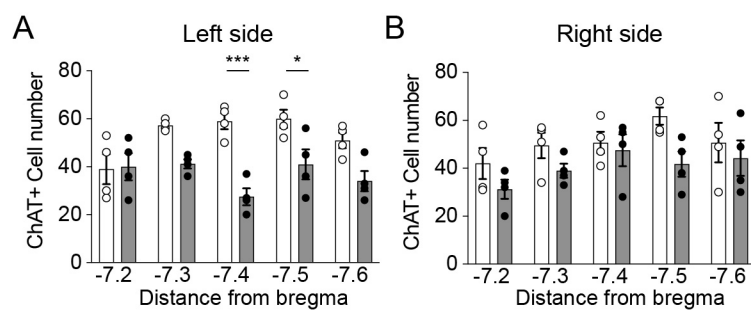

S2 Figure

Supplement: S2 Fig — (A and B) Graphs showing the number of cholinergic neurons on the left and right sides of the DMV in the control and experimental groups. Unpaired t test, *p < 0.05; ***p < 0.001. The data supporting the graphs shown in the figure (S2A and S2B Fig) are available in the S2 Data file. (PDF) [file pbio.3002865.s002.pdf]

A. ChAT<sup>Cre</sup> fed a standard chow diet

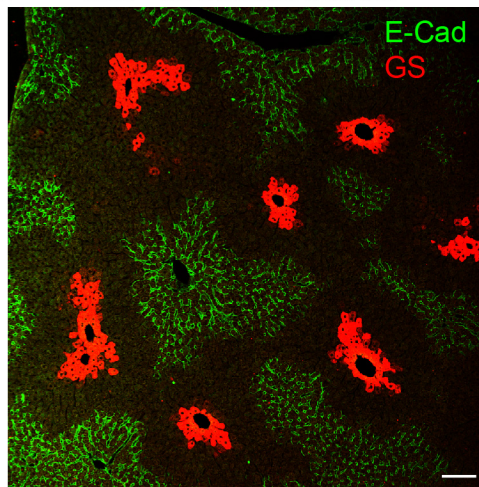

Supplement: S3 Fig — (A) Image of confocal fluorescence microscopy showing the expression of E-Cad and GS in the liver parenchyma. Scale bar, 100 μm. (PDF) [file pbio.3002865.s003.pdf]

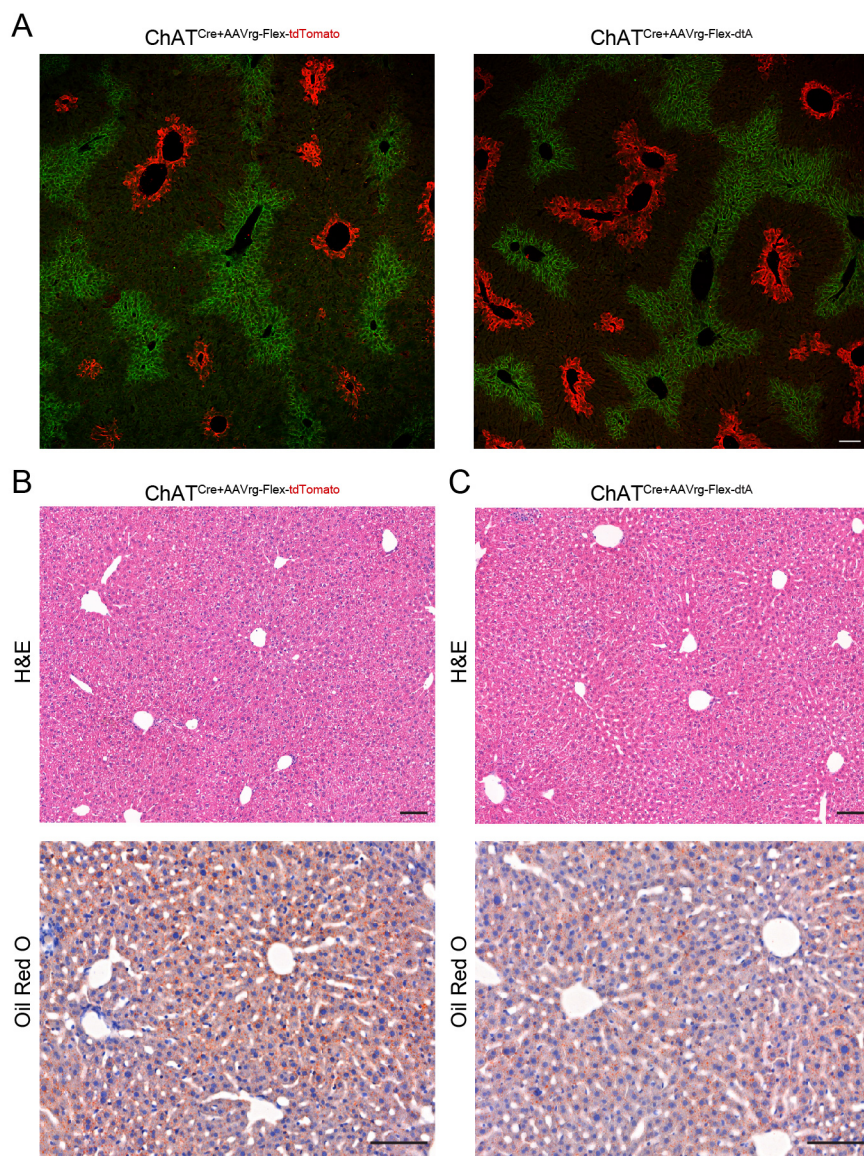

S4 Figure

Supplement: S4 Fig — (A) Macroscopic appearance of the livers of ChATCre female mice receiving AAVrg-FLEX-GFP and AAVrg-FLEX-dtA. E-Cad (green) and GS (red) staining in the control and the experimental groups. Scale bar, 100 μm. (B, C) HE and Oil Red O staining of liver tissues from the control and experimental groups. No histological differences were observed between the groups (upper panels, scale bar, 50 μm; bottom panels, scale bar, 100 μm). (PDF) [file pbio.3002865.s004.pdf]

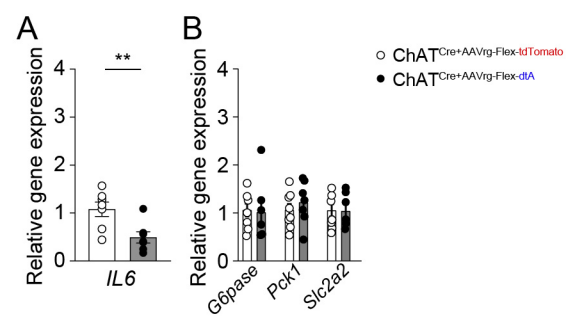

S5 Figure

Supplement: S5 Fig — (A) Relative Il6 mRNA expression in the livers of the control (n = 7 mice) and experimental (n = 7 mice) groups. Unpaired t test, **p < 0.01. (B) Graph showing mRNA expression of the gluconeogenic enzymes in the livers of the control and the experimental groups (control, n = 8 mice; experimental, n = 7 mice). The data supporting the graphs shown in the figure (S5A and S5B Fig) are available in the S2 Data file. (PDF) [file pbio.3002865.s005.pdf]

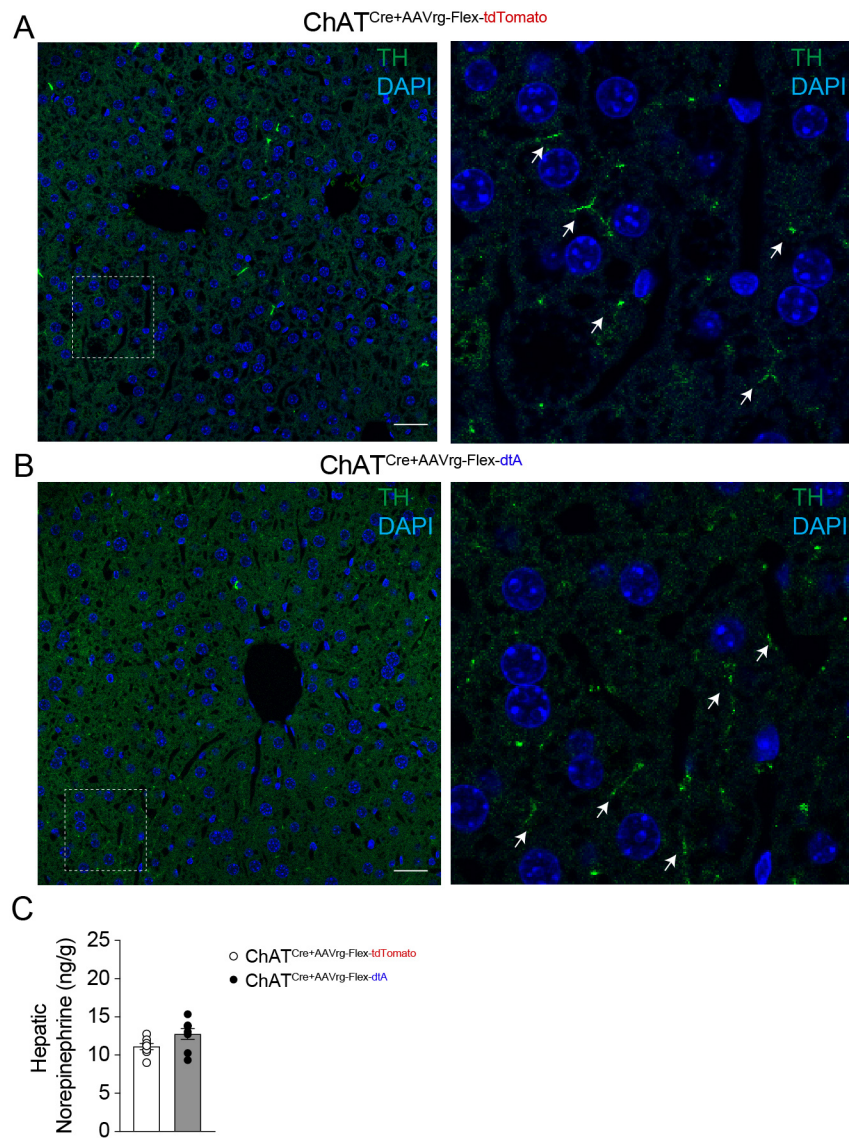

S6 Figure

Supplement: S6 Fig — (A, B) Images of confocal fluorescence microscopy showing TH-positive nerve fibers in the liver parenchyma of ChATCre mice with and without liver-projecting cholinergic neurons (arrows). Scale bar, 30 μm. Right panel: Higher magnification view of the white dotted square. (C) Graph showing hepatic norepinephrine levels in the control (n = 8 mice) and experimental (n = 8 mice) groups. The data supporting the graphs shown in the figure (S6C Fig) are available in the S2 Data file. (PDF) [file pbio.3002865.s006.pdf]

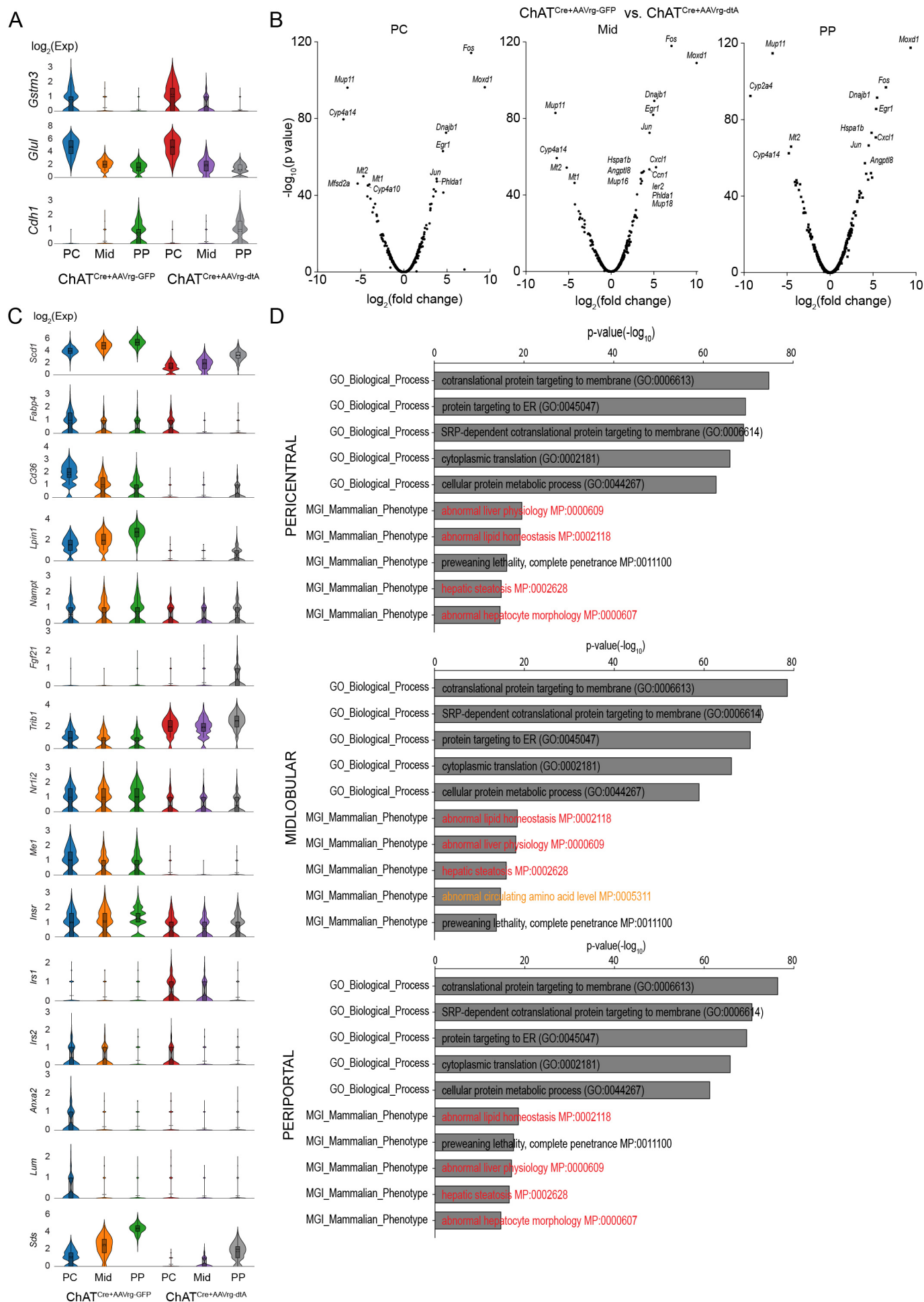

S7 Figure

Supplement: S7 Fig — (A) Violin plots depicting the differential expression of the liver zonation marker genes in the pericentral, midlobular, and periportal areas in the control and experimental groups. (B) Volcano plots illustrate the differences in gene expression across the zonation of the livers in control and experimental mice. (C) Violin plots displaying the enriched genes across the zonation of the livers in control and experimental mice. (D) Plots displaying the top GO terms in biological process and mammalian phenotype across the zonation of the livers in control and experimental mice. The data supporting the graphs shown in the figure (S7A–S7D Fig) are available in the S2 Data file. (PDF) [file pbio.3002865.s007.pdf]

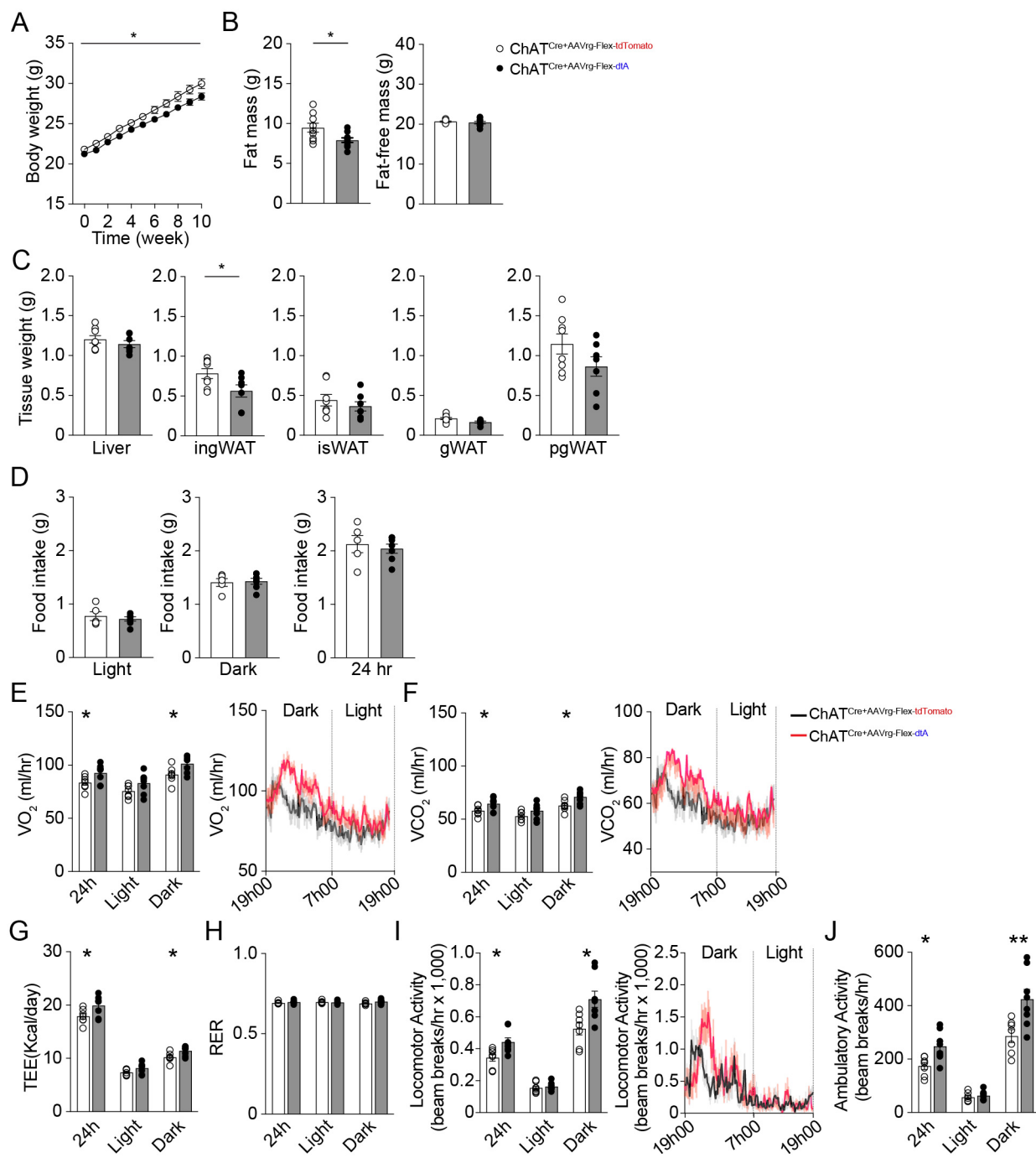

S8 Figure

Supplement: S8 Fig — (A) Graph showing changes in body weight of the control and experimental groups during high-fat diet feeding. There was a significant difference in body weight gain between the groups (control, n = 12 mice; experimental mice, n = 21 mice; two-way ANOVA test, *p < 0.05). (B) Graphs show a significant difference in body fat but not fat-free mass between the 2 groups (fat mass and lean mass, control, n = 9 mice; experimental mice, n = 10 mice, unpaired t test, *p < 0.05). (C) Graphs showing that the experimental mice (n = 7) exhibited a significantly lower ingWAT mass than the control group (n = 8), whereas there was no difference in liver, interscapular WAT (isWAT), gonadal WAT (gWAT), and perigonadal WAT (pgWAT) mass between the groups. Unpaired t test, *p < 0.05. (D) Graphs showing that both groups consumed the same amount of food (control, n = 5 mice; experimental mice, n = 7 mice). (E, F) Summary plots showing VO2 and VCO2 between the groups. A significant difference in VO2 and VCO2 in the dark phase was observed between the groups (control, n = 7 mice, experimental mice, n = 8 mice). Unpaired t test, *p < 0.05. (G, H) Summary plots showing total energy expenditure (TEE) and respiratory exchange ratio (RER) between the groups (control, n = 7 mice, experimental mice, n = 8 mice). The loss of hepatic cholinergic input significantly increased TEE in the dark phase. Unpaired t test, *p < 0.05. (I, J) Summary plots showing total locomotor and ambulatory activities between the experimental groups (control, n = 7 mice, experimental mice, n = 8 mice). Unpaired t test, *p < 0.05. The data supporting the graphs shown in the figure (S8A–S8J Fig) are available in the S2 Data file. (PDF) [file pbio.3002865.s008.pdf]

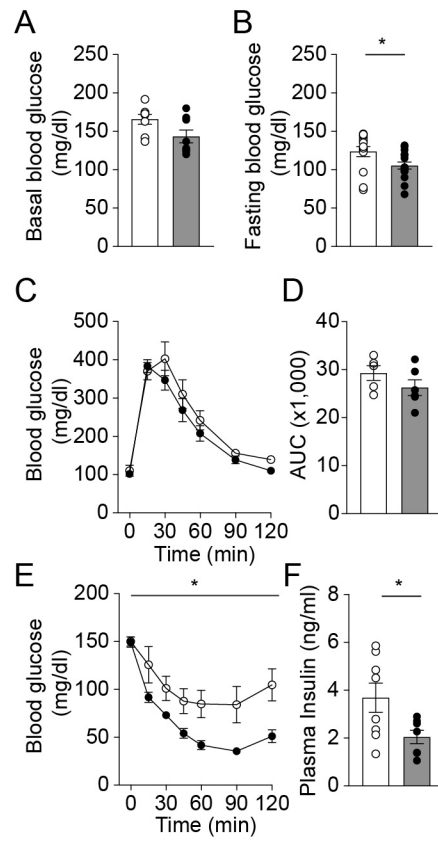

Supplement: S9 Fig — (A, B) Summary graphs showing the basal (non-fasting) and fasting glucose levels in the control (open circle) and experimental (closed circle) groups. Unpaired t test, *p < 0.05. (C, D) Summary graphs showing the i.p. GTT in ChATCre mice with (n = 5 mice) and without (n = 6 mice) liver-projecting cholinergic neurons. AUC: area under the curve. (E) Plot showing the i.p. ITT in ChATCre mice with and without parasympathetic cholinergic neurons innervating the liver (two-way ANOVA followed by Sidak multiple comparisons test, control, n = 6 mice; experimental group, n = 6 mice, F(1, 10) = 6.6, *p = 0.03). (F) Graph showing plasma insulin levels in the controls (n = 8 mice) and the experimental group (n = 7 mice). There was a significant difference in plasma insulin levels between the groups. Unpaired t test, *p < 0.05. The data supporting the graphs shown in the figure (S9A–S9F Fig) are available in the S2 Data file. (PDF) [file pbio.3002865.s009.pdf]

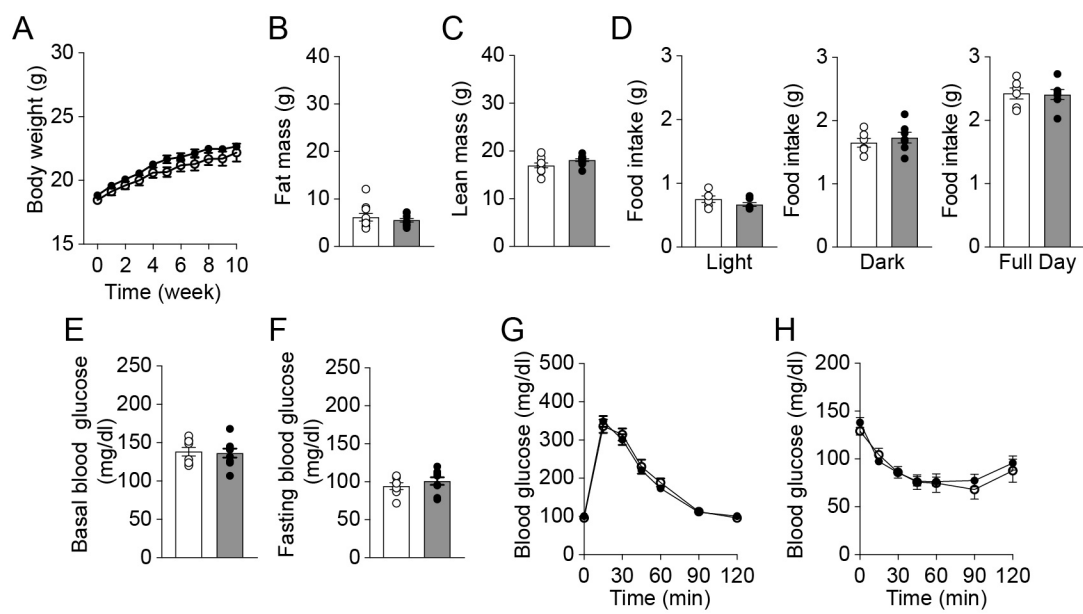

S10 Figure

Supplement: S10 Fig — (A) Graph showing changes in body weight of female ChATCre mice with and without parasympathetic cholinergic innervation to the liver during high-fat feeding. There was no significant difference in body weight gain between the groups (control (open circle), n = 10 mice; experimental (closed circle), n = 11 mice). (B, C) Plots show no differences in body fat and lean mass between the 2 groups (control, n = 10 mice; experimental mice, n = 11 mice). (D) Graphs showing that both groups consumed the same amount of food (control, n = 6 mice; experimental mice, n = 7 mice). (E, F) Summary graphs showing the basal (non-fasting) and fasting glucose levels. There were no significant differences in basal and fasting glucose levels between groups (control, n = 8 mice; experimental mice, n = 9 mice). (G, H) Summary graphs showing the i.p. GTT and i.p. ITT in mice with and without liver-projecting cholinergic neurons (GTT: control, n = 8 mice; experimental mice, n = 9 mice; ITT: control, n = 9 mice; experimental mice, n = 10 mice). No significant differences were observed between the groups. The data supporting the graphs shown in the figure (S10A–S10H Fig) are available in the S2 Data file. (PDF) [file pbio.3002865.s010.pdf]
