# Supplementary material for: The development of hepatic steatosis depends on the presence of liver-innervating parasympathetic cholinergic neurons in mice fed a high-fat diet
Source: PLoS Biol. 2024 Oct 22;22(10):e3002865. doi: 10.1371/journal.pbio.3002865 (PMC11530026; doi:10.1371/journal.pbio.3002865)

Fig4_Raw_image

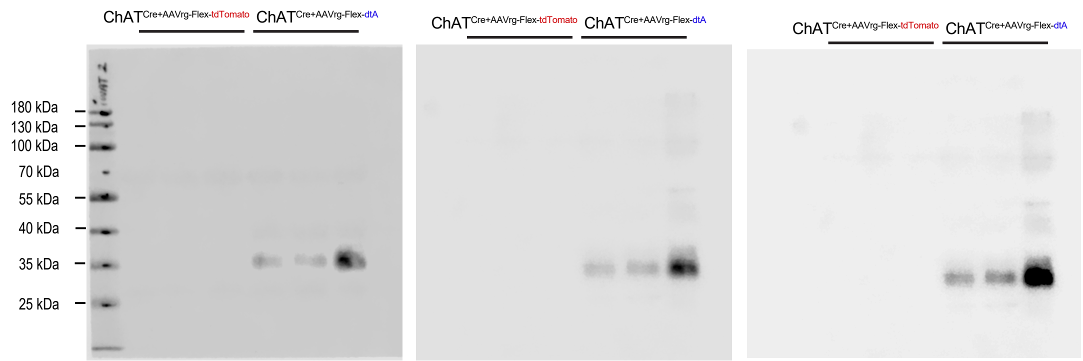

Fig. 4F-Anti-UCP1

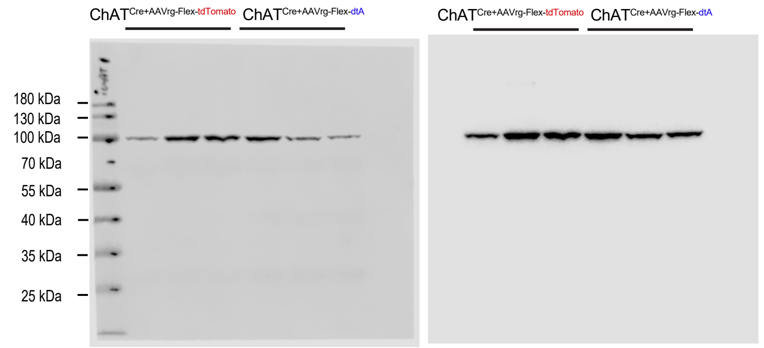

Fig. 4F-Anti-HSP90

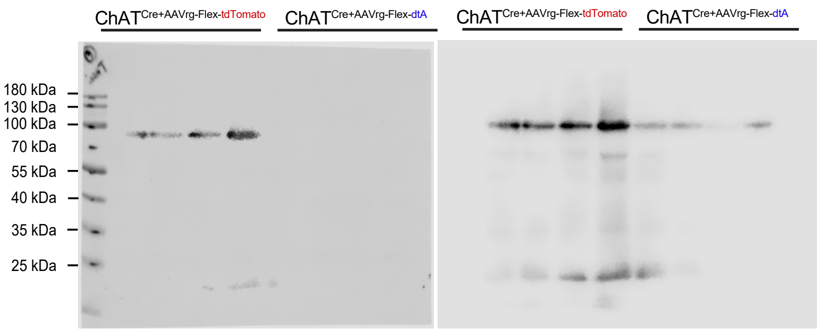

Fig. 4I-Anti-pS660-HSL

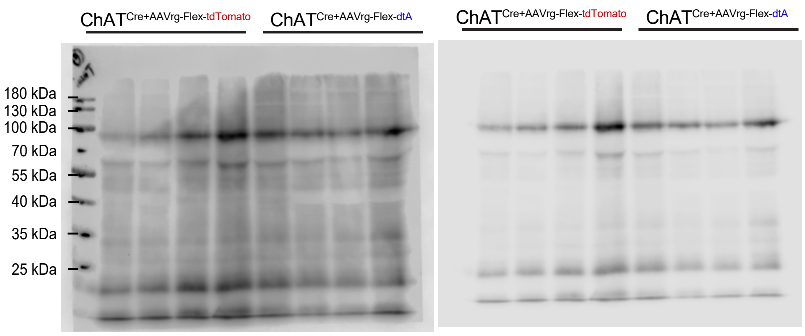

Fig. 4I-Anti-HSL

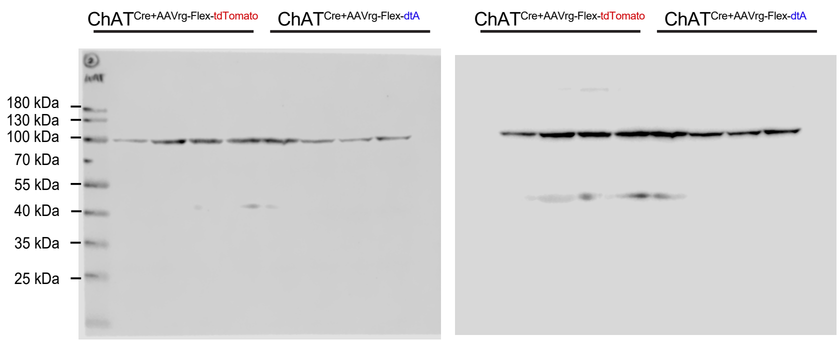

Fig. 4I-Anti-HSP90

Fig6_Raw_image

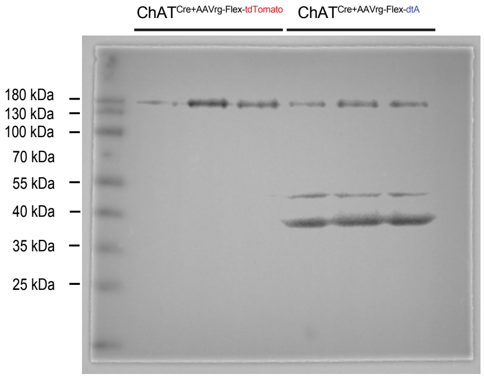

Fig. 6B-Anti-TGR5

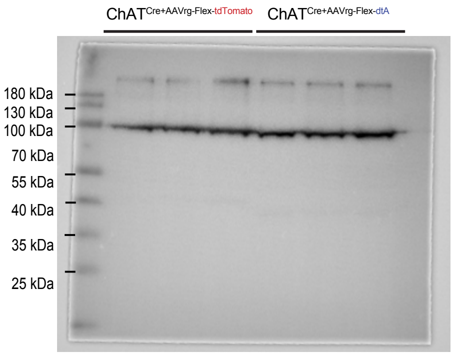

Fig. 6B-Anti-HSP90

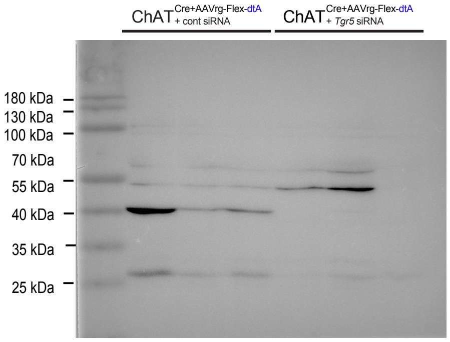

Fig. 6D-Anti-TGR5

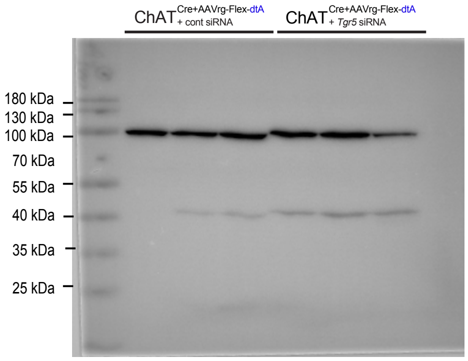

Fig. 6D-Anti-HSP90

Fig7_Raw_image

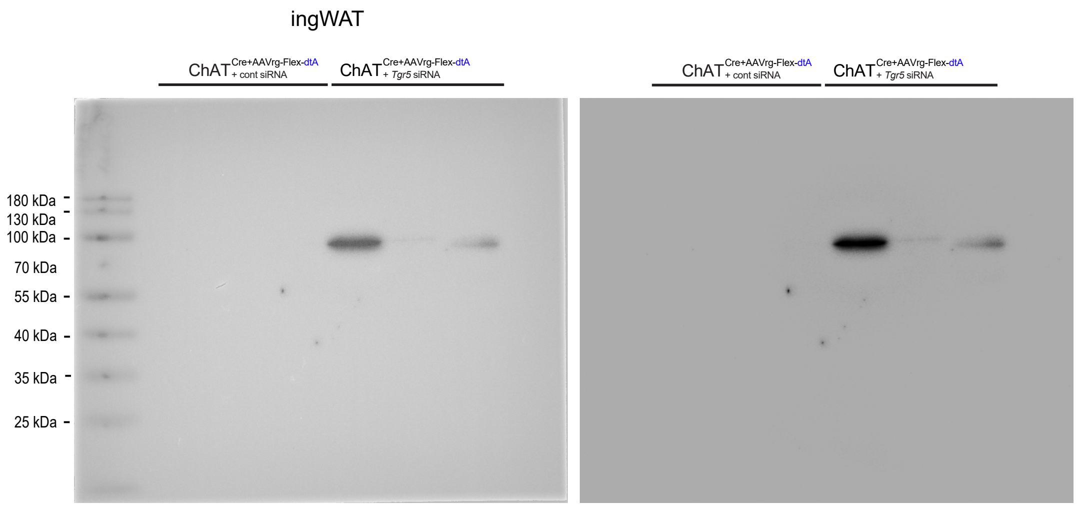

Fig. 7A-Anti-pS660-HSL

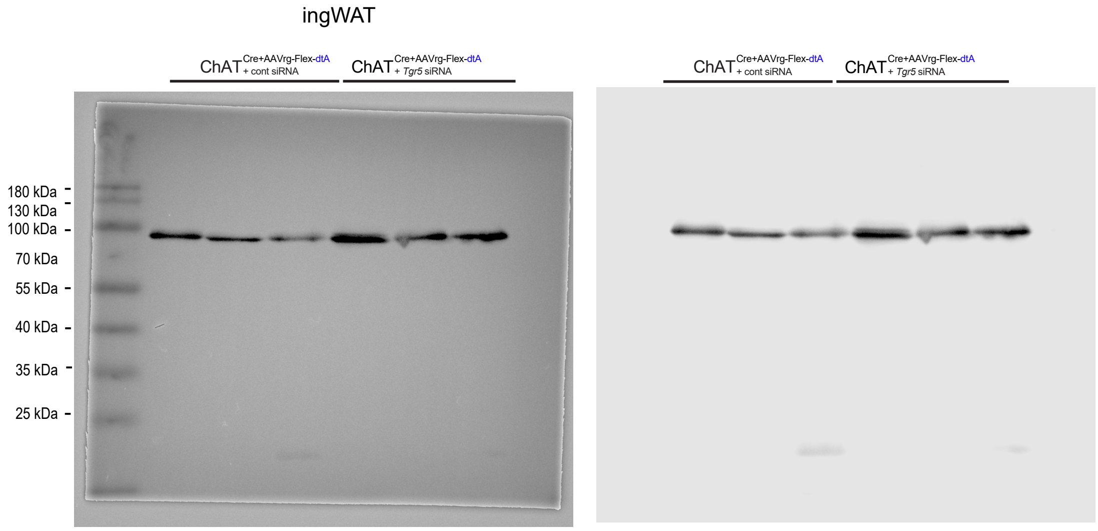

Fig. 7A-Anti-HSL

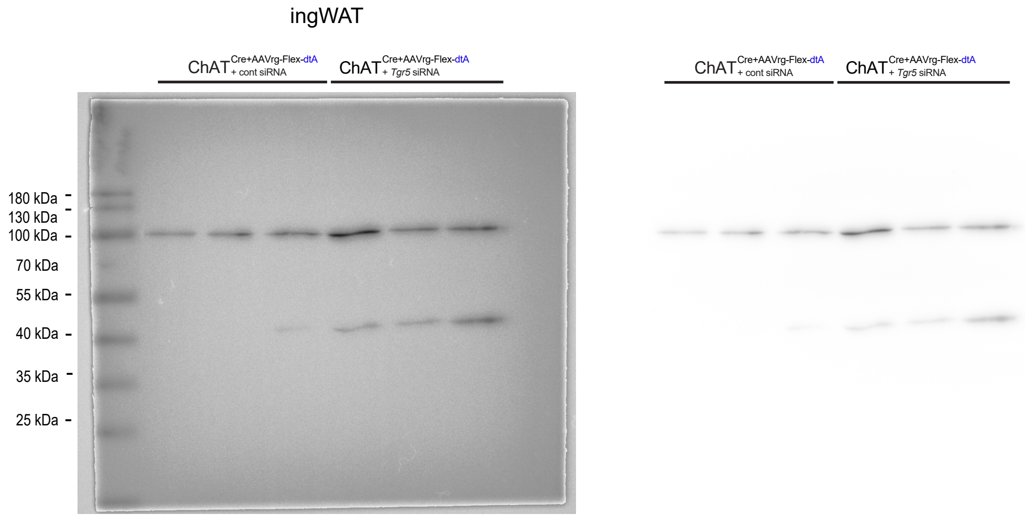

Fig. 7A-Anti-HSP90

S1_Raw_image

S1.Fig. 1B

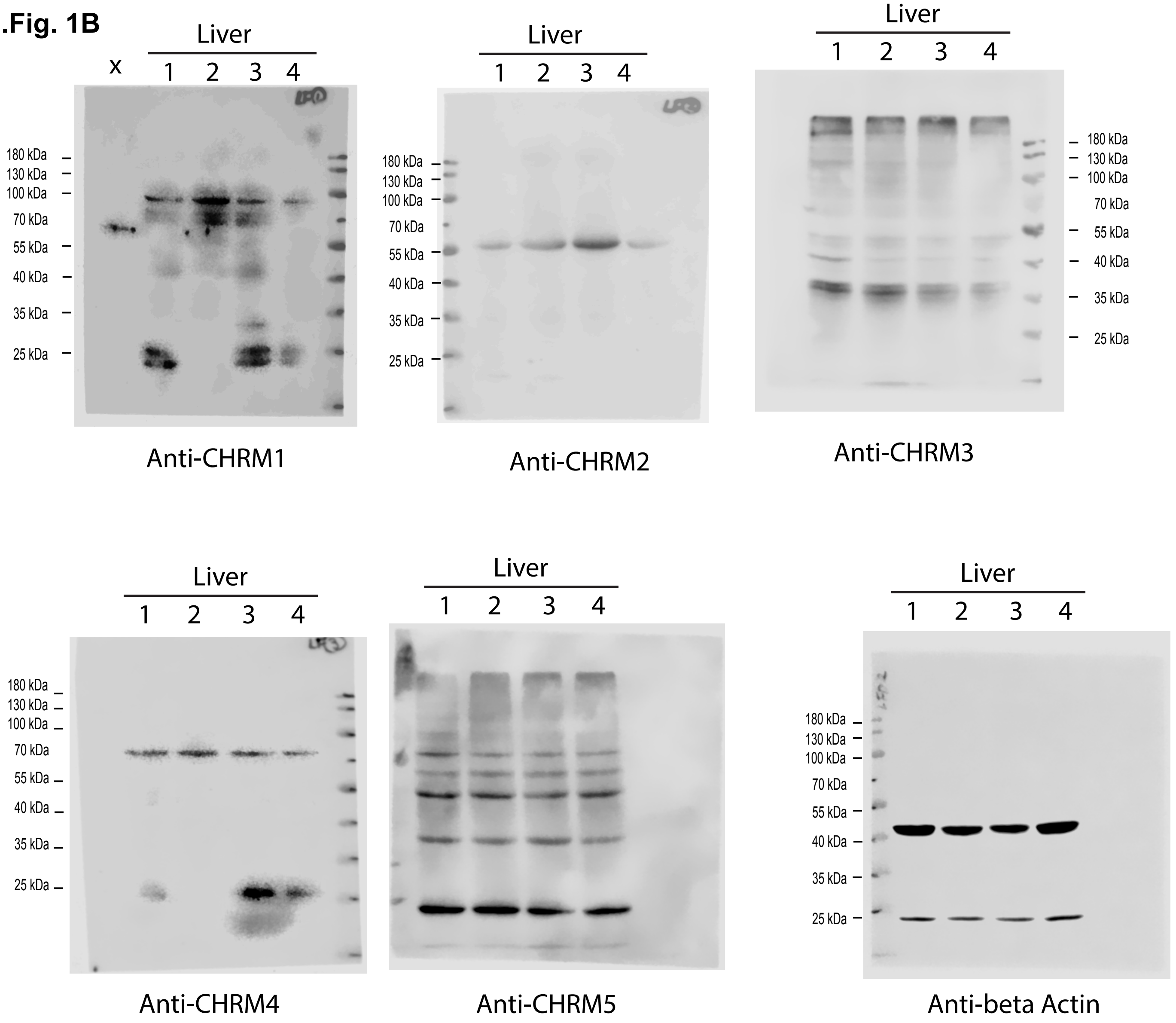

S1.Fig. 1C

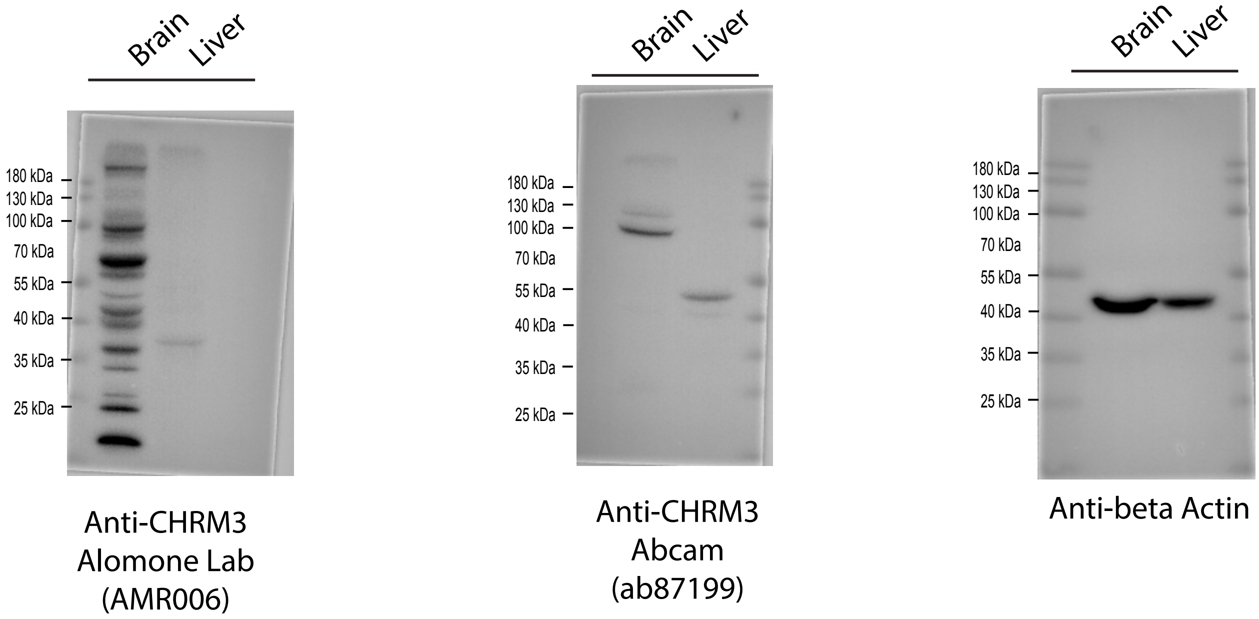

Supplement: S1 Raw Images — (PDF) [file pbio.3002865.s013.pdf]
